# Preoperative elevation of serum C – reactive protein is predictive for prognosis in myeloma bone disease after surgery

**DOI:** 10.1038/sj.bjc.6603329

**Published:** 2006-09-12

**Authors:** A Zahlten-Hinguranage, H Goldschmidt, F W Cremer, G Egerer, T Moehler, D Witte, L Bernd, D Sabo, F Zeifang

**Affiliations:** 1 Department of Orthopedic Surgery, University of Heidelberg, Heidelberg, Germany; 2 Department of Internal Medicine V, University of Heidelberg, Germany

**Keywords:** multiple myeloma, bone, surgery, CRP, prognostic factor

## Abstract

We investigated whether preoperative levels of serum C-reactive protein (CRP) and its correlation with tumour clinicopathological findings adds prognostic information beyond the time of diagnosis in patients with myeloma bone disease (MM) to facilitate the surgical decision-making process. Six hundred and fifty-eight myeloma patients were evaluated retrospectively for surgery. Clinicopathological variables of patients who underwent surgery (*n*=71) were compared between patients with preoperative CRP ⩾6 mg l^−1^ and those with CRP <6 mg l^−1^. Univariate and multivariate analyses were performed to identify prognostic factors after surgery. Patients with an increase of CRP prior to surgery showed inferior survival compared to patients with normal levels. Patients with normal CRP levels at diagnosis but elevations prior to surgery do seem to have a similar unfavourable overall survival (OS) than patients with an increase both, at diagnosis and at surgery. Conversely, patients with normal CRP levels prior to surgery still have the best OS, irrespective of their basic values. Multivariate analysis revealed preoperative CRP levels above 6 mg l^−1^ Lactate dehydrogenase (LDH) above normal, and osteolyses in long weight bearing bones as independent predictors of survival. These findings suggest that in patients with MM serum levels of CRP increase during disease activity and might be significantly correlated with specific disease characteristics including adverse prognostic features such as osteolyses in long weight bearing bones. Thus, preoperative elevated CRP serum levels might be considered as independent predictor of prognosis and could provide additional prognostic information for the risk stratification before surgical treatment in patients with myeloma bone disease.

One of the most important clinical features of multiple myeloma (MM) is the development of lytic bone lesions. Over 70% of patients present with skeletal lesions at diagnosis (Berenson, 2005). Besides systemic treatment, these patients frequently require local radiotherapy, agents such as bisphosphonates, and long-term analgesic medications. Surgery has a supportive role in the management of myeloma and is performed with the intention to surgically stabilise an impending or existing pathologic fracture or an osteolytic deposit.

Although the outcome has improved with a median survival of 4–5 years since the introduction of high-dose therapy (HDT) with peripheral blood stem cell transplantation (PBSCT) and new drugs like Thalidomide and Bortezomib, aggressive features of myeloma bone lesions have significantly contributed to a poor outcome (Durie *et al*, 2003; Tricot *et al*, 2004; Morgan and Davies, 2005; Rajkumar and Kyle, 2005; Glasmacher *et al*, 2006).

In general, the decision to proceed with surgery is complex and depends on survival estimates based on established prognostic factors and largely on the clinical status of the patient. For example, in cases of vertebral fractures, for patients with a life expectancy of only a few months, surgery is not mandatory in the absence of neurological deficits, if advantages offered by other forms of treatment exist. Therefore, for identifying those patients at risk for early death and for selecting appropriate surgical interventions if longer survival is expected, it would be extremely valuable for orthopedic surgeons to find a marker of disease aggressiveness at the time of surgery that is easy to asses. In addition to the prognostic value of baseline biological markers including high levels of *β*2-microglobulin (B2-M), lactate dehydrogenase (LDH), and serum albumin (Bataille *et al*, 1992), elevations of serum C-reactive protein (CRP) have been consistently associated with a poor prognosis in myeloma (Tienhaara *et al*, 1994; Kaneko *et al*, 2002; Terpos *et al*, 2003). Several studies regarding the role of preoperative CRP as a predictive indicator for the malignant potential and prognosis in various other cancers have been published (Nozoe *et al*, 1998; Nakanishi *et al*, 2002; Shimada *et al*, 2003; Hashimoto *et al*, 2005). However, the prognostic significance based on preoperative CRP has not been clarified for myeloma patients, yet. Based on preliminary results (Zeifang *et al*, 2005), our intention was to describe the surgical outcome of patients with myeloma bone disease and to investigate whether preoperative CRP levels and its correlation with tumour clinicopathological findings add prognostic information to patients with myeloma bone disease in order to facilitate the surgical decision-making process.

## METHODS

### Patients

A total of 658 myeloma patients who were treated with high-dose therapy (HDT) and autologous peripheral blood stem cell transplantation (PBSCT) according to clinical trial protocols (Goldschmidt, 2003) were reviewed retrospectively. Patients were conditioned with either one (*n*=199) or two courses of melphalan 200 mg m^−2^ (*n*=379) or with a combination of melphalan 140 mg m^−2^ and total body irradiation (TBI) (*n*=80). Eighty-four patients (12.4%) underwent surgery for their osteolyses. Of these, 13 patients were treated according to clinical trial protocol with melphalan 140 mg m^−2^ and TBI, 24 patients with a single course melphalan 200 mg m^−2^, and 47 patients with two courses of melphalan 200 g m^−2^. The complete and partial response rates were 29.6 and 70.4%. All patients received supportive care measures for the treatment of anaemia and hypercalcaemia as well as long-term bisphosphonate therapy. The state of disease prior to surgery was retrospectively assessed.

The indication for surgical treatment at the extremities included lesions with elevated fracture risk according to Mirels' Scoring System (Mirels, 1989) or pathological fractures. By definition an impending pathological fracture risk (>30%) is attributed to scores of eight or higher. Indication for surgical intervention at the vertebral column included progressive neurological impairment according to the Grading System of Frankel (1969) assessing sensory and motor function or severe vertebral body lesions. Appropriate laboratory tests as well as a physical examination by an internist, anaesthetist and the surgeon were routinely drawn on the day before surgery for all patients in order to evaluate the surgical risk and to identify patients with infections. Contraindications to surgery were any signs and symptoms of preoperative infections.

The median age at the time of initial treatment was 55 years, with a range of 30–70 years. Follow-up ranged from 8 to 179 months, with a median of 45 months. At the time of analysis, 29 patients (34.5%) had died.

### Parameters

In addition to demographic data, the patients were assessed for monoclonal protein isotypes, number and anatomic site of bone lesions, state of disease prior to surgery, surgery prior to initial treatment, and prior local radiation. Biochemical features prior to surgery including CRP, B2-M, LDH, serum albumin, haemoglobin and creatinine were collected from medical records. As elevations of CRP above 6 mg l^−1^ may indicate a poorer prognosis, patients with a rise of CRP above this level were assigned to the CRP positive group.

### Statistical analysis

Descriptive statistics were performed for all variables and expressed as means±SD. Comparisons between the two groups were calculated with the Mann–Whitney *U*-test for quantitative data, with χ^2^ or fisher exact test for qualitative data. The end point of interest was survival time, defined as the time from surgery to death or last follow-up date. In addition to demographic data (age, sex), independent variables consisted of clinical variables (number and anatomical site of bone lesions) and medical variables (tumour state prior to surgery, monoclonal protein isotypes, preoperative CRP, LDH, haemoglobin and creatinine, prior local radiation, and surgery prior to initial treatment). Normal serum LDH was 100 to 190 U l^−1^ for patients' age ⩽60 years and 110 to 210 U l^−1^ for patients' age >60 years.

The overall survival rates after surgery were calculated according to Kaplan–Meier. Any significant differences were determined with the log rank statistic. Univariate and multivariate Cox stepwise proportional hazard models were used to identify independent predictors of overall survival. Only those variables found to be significant (*P*<0.10) in the univariate model were entered into the multivariate model. A significance level of 0.10 was used for entry and retention of variables in the stepwise multivariate model. *P*-values <0.05 were considered significant. As the entire set of laboratory data was not available for 13 patients, those were excluded from this study. In total, 71 patients with complete data were included in this retrospective analysis. The statistical software used for the calculation was SPSS (Standard Version 10.0, SPSS, Inc., Chicago, IL, USA).

## RESULTS

Of 71 surgically treated patients with myeloma bone disease, evaluated for CRP elevation prior to surgery, 26 (36.6%) were assigned to the negative group and 45 (63.4%) to the positive group. The median CRP levels were 2.7 mg l^−1^ (range 1.0–4.0) in the negative group and 9.2 mg l^−1^ (range 6.0–76.4) in the positive group, respectively. With an average of 13 months, the time intervals between the diagnosis and surgery were higher for patients with increased CRP levels compared to 6.2 months for patients with normal values. The state of the disease prior to surgery is shown in Table 1. While serum albumin and haemoglobin levels in the CRP positive group were significantly lower compared with the CRP negative group (*P*=0.019 and *P*=0.055, respectively), B2-M, LDH and serum creatinine were well balanced between the study groups (*P*=0.678, *P*=0.123, and 0.554, respectively). The key prognostic variables of the two groups are summarised in Table 2.

Clinically relevant osteolytic lesions were located in the vertebral column in 42 (59.2%), ribs in two (2.8%), proximal humerus in one (1.4%), humerus shaft in eight (11.3%), proximal femur in seven (9.9%), femur shaft in six (8.5%) and each one in the tibia shaft, ulna shaft, cranial bone, clavicula and jaw, respectively.

Vertebral tumours were excised through anterior and/or posterior approach, combined with a stabilising procedure in 41 (97.6%) cases, or kyphoplasty in one (2.4%) case. Either intramedullary rod fixation (*n*=16; 69.5%) or endoprosthesis (*n*=7; 30.5%) were inserted to give support for weight bearing bones. Five tumours were excised through local tumour resection without stabilisation. In all 25 patients (35.2%) underwent local radiation prior to surgery. In 34 (47.9%) cases, orthopedic surgery had to be performed before the onset of initial treatment. With 9.8% (7/71) the observed complication rate following surgery was low. The median age at surgery was 56 years, with a range from 30 to 68 years. After a median follow-up of 57 months (4–132 months), 29 patients (40.8%) had died. The 1-, 3-, and 5-year OS rates were 92.7, 62.1 and 47.9%, respectively. The peri- and postoperative mortality rate within 30 postoperative days was zero in this group.

### Overall survival after surgery

**Figure 1:**
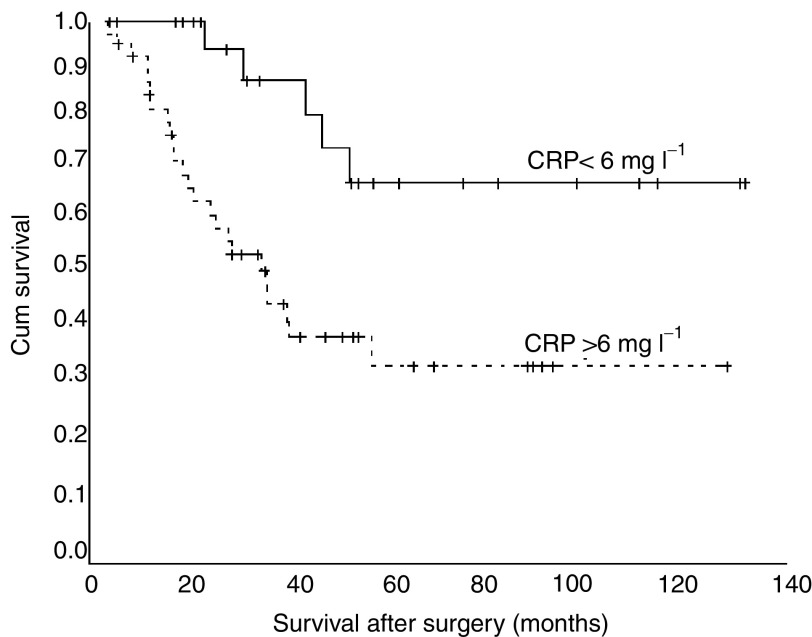
Overall survival rates after surgery were significantly higher for patients with a CRP level <6 mg l^−1^ prior to surgery compared to patients with CRP >6 mg l^−1^ (*P*=0.0046).

Patients with a lower CRP level (<6 mg l^−1^) prior to surgery had a better OS after surgical intervention with a median survival of 58.0 months than patients with a rise above this level (median survival 35.9 months) (*P*=0.0046). Overall survival estimates at 1, 3 and 5 years were 100.0, 89.2 and 70.1%, respectively, *vs* 88.6, 48.0 and 36.4%, respectively (Figure 1).

### Predictors of mortality

In univariate Cox analysis, significantly poorer predictive values were found for or a rise of CRP ⩾6 mg l^−1^ prior to surgery (*P*=0.008), LDH above normal (*P*=0.027), serum creatinine ⩾2 mg dl^−1^ (*P*=0.036), and lytic lesions in long weight bearing bones (*P*=0.001). Moreover, the state of disease prior to surgery was found to have prognostic influence by univariate analysis, too (*P*=0.026). In particular, patients with HDT and PBSCT in relapse prior to surgery were 3.3 times more likely to die than those patients whose surgery was performed prior to systemic therapy. Subsequent, surgery prior to initial treatment was found to be a positive prognostic factor for survival in patients with myeloma bone disease (*P*=0.006). The prognostic value of each factor analysed by univariate analysis is listed in Table 3.

**Figure 2:**
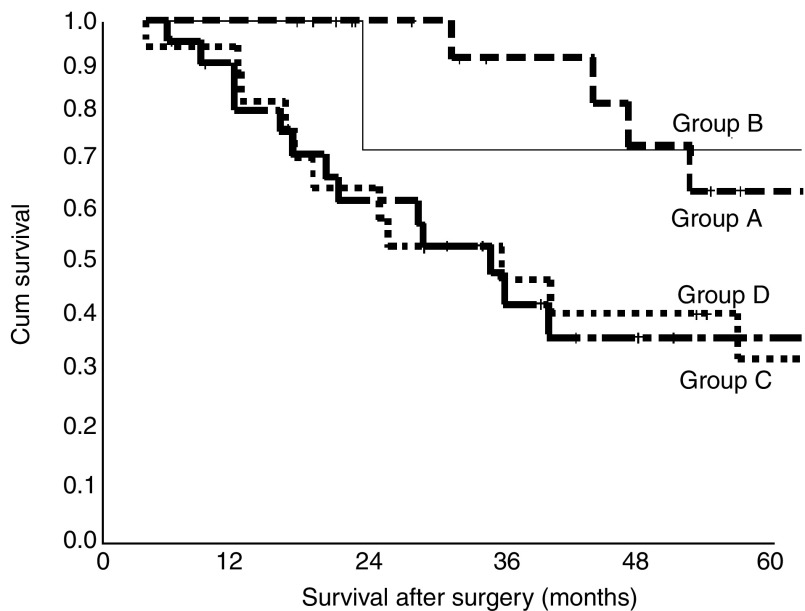
Overall survival rates after surgery per rise of CRP. Number of patients: Group *A*=21; Group *B*=5; Group *C*=20; Group *D*=25.

The effect on survival of the dynamic of serum CRP over time was evaluated (Table 4). Patients were classified according to their CRP levels at diagnosis and at surgery. Survival was greater for patients with normal preoperative CRP levels compared to those patients with elevations irrespective of their initial CRP values (*P*=0.0446) (Figure 2).

The results of multivariate analysis using the stepwise Cox proportional hazards regression model are shown in Table 5. In particular, preoperative serum CRP levels above 6 mg l^−1^ (HR=2.721; *P*=0.048) and osteolyses in long weight bearing bones (HR=0.476; *P*=0.058) remained as independent predictors of mortality.

## DISCUSSION

Although elevations of C-reactive protein (CRP) serum levels in newly diagnosed patients with myeloma bone disease have been consistently associated with a poor prognosis (Bataille *et al*, 1992; Tienhaara *et al*, 1994; Durie *et al*, 2003) the significance of preoperative CRP levels have not been clarified as of prognostic importance in myeloma patients, yet, and was, for the first time, subject of this study.

The major finding of this investigation is the inferior survival of patients with myeloma bone disease in whom CRP was elevated prior to surgery. This result replicates that of previous research regarding the role of CRP as a predictive indicator for the malignant potential and prognosis in various other cancers and suggests several possibilities: (1) preoperative elevations of CRP indicate a poor outcome in myeloma patients, or (2) bone involvement needing surgery is a significant adverse factor.

Alterations of serum CRP is a common feature in patients with malignancies which has been found to be adversely prognostic, with patients having elevated CRP levels (Nozoe *et al*, 1998; Nakanishi *et al*, 2002; Shimada *et al*, 2003; Hashimoto *et al*, 2005). Growing evidence suggests prognostic importance because increased CRP levels are associated with the production of cytokines from tumour cells (Kurzrock, 1997; Legouffe *et al*, 1998, Yamamura *et al*, 1998). For myeloma it is well known that interleukin-6 (IL-6) plays a crucial role in the cytokine network, regulating the growth and survival of myeloma cells and stimulating the acute-phase protein synthesis, notably C-reactive protein (CRP) (Lauta, 2003). Several authors investigating serum IL-6 in relation to acute-phase reactants and survival in myeloma patients report not only significantly shorter survival for those with higher concentrations of IL-6 compared with patients who had normal concentrations (Ludwig *et al*, 1991; Pulkki *et al*, 1996; Papadaki *et al*, 1997; Stasi *et al*, 1998), but also demonstrate a strong positive correlation between IL-6 and CRP (Tienhaara *et al*, 1994; Pelliniemi *et al*, 1995; Kyriakou *et al*, 1997; Biro *et al*, 1998; Alexandrakis *et al*, 2003). As an assessment of infection status at the time of surgery was made, CRP rather appears to act as a surrogate marker for IL-6 activity and proliferative status of bone marrow plasma cells and has been regarded as a powerful prognostic marker in patients with multiple myeloma, than a sign for preoperative infectious states.

Our results extend previous observations regarding the role of CRP as an independent prognostic marker in multiple myeloma, indicating that CRP has predictive power for patients with myeloma beyond the time of diagnosis. In the present study it could be shown that a raised preoperative CRP concentration was strongly associated with a shorter survival. Patients with elevated CRP levels were 2.72 times more likely to die than patients with normal values. This difference in survival cannot be attributed to response to HDT and PBSCT as the best response rates were similar in both groups. Moreover, the different states of disease prior to surgery failed to remain prognostic significant in multivariate analysis. Although the small number of cases precludes definitive conclusion, it could be demonstrated that patients with normal CRP levels at diagnosis but elevations prior to surgery do seem to have a similar unfavourable overall survival than patients with an increase both, at diagnosis and at surgery. Conversely, in this study population, patients with normal CRP levels prior to surgery had the best overall survival, irrespective of their basic values.

Besides a relation with inferior survival, the current data further showed that the serum CRP levels were inversely correlated with serum albumin. This specific feature of high-serum CRP concentration and hypoalbuminemia in some myeloma patients has been addressed earlier with regard to disease activity (Alexandrakis *et al*, 2003; Durie *et al*, 2003). In contrast to high preoperative CRP levels, we did not find B2-microglobulin as predictive parameter. B2-microglobulin is considered to reflect the tumour load. We hypothesise that in this subgroup of MM patients with clinically relevant osteolytic lesions, a high tumour load can be assumed anyway, while the malignant potential is better indicated by CRP as surrogate marker for IL-6 levels.

Moreover, while B2M is a dominant prognostic factor for survival at diagnosis, its prognostic relevance has not been shown during the course of the disease, yet (San Miguel *et al*, 2004). On the other hand, in accordance with the literature (Nachbaur *et al*, 1991; Pelliniemi *et al*, 1995) preoperative CRP concentrations were adversely correlated with one of the most significant prognostic variables of the disease, haemoglobin level. Consistent with previous reports (Hannisdal *et al*, 1990; Zeifang *et al*, 2005), lytic bone lesions were found to be important for prognosis if they were located in the long bones, too. It is reasonable to assume that bone involvement needing surgery might be a significant adverse factor since myeloma cells first expand in the bone marrow infiltrating the axial skeleton, and with increased cellular proliferation they cause extensive bone destruction and osteolyses in long weight bearing bones. The higher time intervals between the diagnosis and surgery for patients with increased CRP levels might support this hypothesis. However, based on the total of 658 myeloma patients, the 1-, 3- and 5-year OS estimates from diagnosis were 98.3, 83.9 and 65.0% for patients without surgery and 98.5, 78.0 and 55.4%, respectively, for patients undergoing surgical intervention. When stratified for serum CRP concentration patients with lytic lesions in the long bones had significantly lower 1- and 3-year overall survival estimates with preoperatively increased CRP levels than patients with normal values (75.3 and 21.9 *vs* 100.0 and 75.0%, respectively). This provides additional evidence that more or less aggressive features of myeloma bone lesions exists. However, the underlying molecular mechanism of bone destruction and tumour progression is still a field of extensive research and essential for the interpretation of this adverse prognostic feature. Nevertheless, taken into account the higher portion of patients requiring prior radiotherapy and osteolyses in long bones, despite inferior survival surgery is still needed in patients with myeloma bone disease, even with high CRP levels, to stabilise an impending or existing pathological fracture, or to excise an osteolytic deposit as an adjunct to chemotherapy and mainly for symptom control.

## CONCLUSION

Taken together, these findings suggest that in patients with multiple myeloma, serum levels of CRP increase during disease activity and might be significantly correlated with specific disease characteristics including adverse prognostic features such as osteolyses in long weight bearing bones. Thus, preoperative elevated CRP serum levels should be considered as an independent predictor of prognosis beyond diagnosis and might provide additional prognostic information for the risk stratification before surgical treatment in patients with myeloma bone disease.

## Figures and Tables

**Table 1:** Patients' state of disease prior to surgery

**State**	**Criteria**	**No. of patients (%)**
1	Prior to HDT with PBSCT	59 (83.1)
2	After HDT with PBSCT in relapse	7 (9.9)
3	After HDT with PBSCT in remission	5 (7.0)
4	After HDT with PBSCT during treatment of relapse	None

HDT=high dose therapy; PBSCT=peripheral blood stem cell transplantation.

**Table 2:** Key prognostic variables in patients with elevations in c-reactive protein (CRP) prior to surgery

**Variable value**	**CRP <6 mg l^−1^ (*n*=26)**	**CRP ⩾6 mg l^−1^ (*n*=45)**	***P*-value**
Age (yrs)	53.5±8.4	54.7±9.5	0.572
Gender (male:female ratio)	17 : 9	27 : 18	0.653
⩾3 lytic lesions	18 (69.2%)	33 (73.3%)	0.711
			
*M component type*			
Bence Jones or IgD	6 (23.1%)	16 (35.6%)	0.273
*β*2-Microglobulin (mg l^−1^)	2.68±1.4	2.54±0.15	0.678
Serum albumin (g dl^−1^)	39.2±5.8	35.8±5.9	0.019*
LDH (U l^−1^)	168.4±48.1	192.4±70.8	0.123
Hemoglobin (g dl^−1^)	12.7±1.5	11.9±1.6	0.055*
Ca^2+^ (mmol l^−1^)	2.35±0.2	2.47±0.4	0.109
Serum creatinine (mg dl^−1^)	0.82±0.2	0.86±0.3	0.554
			
*Best response to HDT*+*PBSCT*			
Complete response (CR)	9 (34.6%)	12 (26.7%)	0.429
Partial response (PR)	17 (65.4%)	33 (73.3%)	0.480
Prior local radiation	6 (23.1%)	19 (42.2%)	0.104
Surgery prior to initial treatment	15 (57.7%)	19 (42.2%)	0.209
Osteolyses in long bones	6 (23.1%)	19 (42.2%)	0.104
Postoperative complications	2 (7.7%)	5 (11.1%)	0.642

Values shown are the mean±s.d.

* Statistically significant at the 0.05 level.

**Table 3:** Hazard ratios from the univariate Cox PH model for surgically treated patients with myeloma bone disease

**Covariate**	**Coefficient (bi)**	**HR [exp(bi)]**	**95% CI**	***P*-value**
Age	0.001	1.001	0.96–1.04	0.949
Gender	0.146	1.158	0.54–2.50	0.709
Preoperative CRP (<6 *vs* ⩾6 mg l^−1^)	1.305	3.687	1.40–9.70	0.008*
⩾3 lytic lesions	0.070	1.073	0.48–2.42	0.866
Bence Jones or IgD	1.175	1.191	0.95–1.48	0.124
State of disease prior to operation				0.026*
Before HDT and PBSCT				
In relapse	1.205	3.337	1.33–8.37	0.010*
In remission	−0.599	0.550	0.74–4.09	0.559
*β*2-Microglobulin (<3.5 *vs* >3.5 g dl^−1^)	−0.988	0.372	0.09–1.57	0.178
LDH (above normal *vs* normal)	0.824	2.280	1.09–4.73	0.027*
Anaemia (Hb <10 *vs* ⩾10 g dl^−1^)	0.399	1.491	0.35–6.39	0.590
Serum creatinine (⩾2 *vs* <2 mg dl^−1^)	2.266	9.644	1.16–80.1	0.036*
Serum albumin (<3.5 *vs* ⩾3.5 mg l^−1^)	0.455	1.577	0.75–3.31	0.228
Prior local radiation (yes *vs* no)	0.312	1.367	0.65–2.87	0.408
Surgery prior to initial treatment (yes *vs* no)	−1.105	0.331	0.15–0.73	0.006*
Osteolyses in long bones (yes *vs* no)	1.195	3.303	1.59–6.89	0.001*

HDT=high dose therapy; PBSCT=peripheral blood stem cell transplantation.

* *P*⩽0.05.

**Table 4:** Effect of dynamics in CRP

**Group**		**No. of patients**	**No. of patients alive**	**Median survival (months)**	**CI months**
A	Normal CRP at diagnosis and at surgery	21	17	NA^a^	NA
B	Elevated CRP at diagnosis but normal at surgery	5	4	NA^a^	NA
C	Normal CRP at diagnosis but elevated at surgery	20	9	39.9	13.1–66.7
D	Elevated CRP at diagnosis and at surgery	25	12	35.9	22.4–49.4

a Not available.

**Table 5:** Hazard ratios from the multivariate Cox PH model for surgically treated patients with myeloma bone disease

**Covariate**	**Coefficient (bi)**	**HR [exp(bi)]**	**95% CI**	***P*-value**
Preoperative CRP (<6 *vs* ⩾6 mg l^−1^)	1.001	2.721	1.01–7.33	0.048
Osteolyses in long bones	−0.742	0.476	0.22–1.03	0.058
